# Enhancing deprescribing: A qualitative understanding of the complexities of pharmacist‐led deprescribing in care homes

**DOI:** 10.1111/hsc.14099

**Published:** 2022-11-06

**Authors:** Linda Birt, David J. Wright, Jeanette Blacklock, Christine M. Bond, Carmel M. Hughes, David P. Alldred, Richard Holland, Sion Scott

**Affiliations:** ^1^ School Allied Health Professionals University of Leicester Leicester UK; ^2^ Allied Health Professionals University of Leicester Leicester UK; ^3^ Institute of Applied Health Sciences University of Aberdeen Leicester UK; ^4^ School of Pharmacy Queen's University Belfast Leicester UK; ^5^ School of Healthcare University of Leeds Leicester UK; ^6^ Leicester Medical School University of Leicester Leicester UK

**Keywords:** Care home staff, deprescribing, GP, Medicines management, older people, over prescribing, primary care

## Abstract

The English National Overprescribing Review identified that older people often take eight or more medicines a day. The report recommended pharmacists in primary care should take responsibility for addressing polypharmacy. Overprescribing is a safety concern in care homes as approximately half of older care home residents are prescribed at least one medicine that is unnecessary or now harmful. This predisposes them to adverse outcomes including hospitalisation and mortality. Deprescribing is the planned activity of stopping or reducing a medicine that may no longer be appropriate. Deprescribing, when performed by a pharmacist, is a multidisciplinary activity requiring close communication with general practitioners (GPs) and care home staff. A recently completed trial that integrated pharmacists with prescribing rights into older peoples' care homes found significant variation in proactive deprescribing activity. The aim of the current study was to specifically explore beliefs and practices of deprescribing in care homes. A qualitative approach was adopted to examine individual, social and contextual factors that acted as enablers and barriers to pharmacist deprescribing in care homes. Semi‐structured interviews were conducted with participants of the previous study (16 pharmacists, 6 GPs and 7 care home staff from Northern Ireland, Scotland and England). Using thematic analysis, we identified two themes: (a) *Structures and systems affecting deprescribing*, that is the context in which deprescribing happened, including team involvement and routine practices in GP surgeries and care homes; (b) *Balancing risks when deprescribing*, that is the perception of individual risk and social barriers were mitigated by understanding the medical background of residents. This supported the clinical understanding that risks from overprescribing were greater than risks from deprescribing. While deprescribing can involve all health professionals in the primary care team, these results suggest the pharmacist is well placed to lead the process; by having both clinical competence and professional willingness to drive this activity forward.

**What is known about this topic:**

1. Older people in care homes are frequently prescribed medicines that are unnecessary or that may now be harmful.
2. Unnecessary or harmful medicines predispose care home residents to avoidable morbidity, hospitalisation and mortality.
3. There is limited policy guidance on who should have responsibility for medication review and potentially stopping medicines.

**What this paper adds:**

1. Pharmacist‐independent prescribers in primary care are optimally placed to support structured medication reviews, initiate and complete deprescribing in care homes.
2. Deprescribing is most likely to happen when pharmacists work effectively together with care home staff and the wider primary care team.
3. Understanding the resident's full clinical history helps to inform good deprescribing decisions.

## 1 INTRODUCTION

Older people in care homes are often subject to complex medication regimens and polypharmacy, which is usually defined as receiving five or more concurrent medicines a day (Masnoon et al., 2017), some of which may not be clinically necessary (NHS Scotland, 2022). It is important to address polypharmacy because age‐associated senescence alters older people's response to medicines, which increases the chances of a medication becoming unnecessary or harmful (Cantlay et al., 2016). While some longer‐term conditions necessitate prescribing of multiple medicines, studies have found that up to 50% of care home residents are prescribed at least one medicine that is unnecessary or now harmful (Duerden et al., 2013). Exposure to these medicines increases the risk of falls and other adverse events leading to morbidity, hospitalisation and mortality (Leelatanok et al., 2017; Blalock et al., 2020). This is particularly so with medicines acting on the central nervous system, such as anticholinergics and sedatives, which are frequently prescribed to help manage behavioural symptoms in people living with dementia (Harrison et al., 2019).

In England, the National Overprescribing Review found older people are at risk from polypharmacy. While the number of medicines taken each day increases after the age of 40, over half of the people aged over 80 take eight or more medicines per day (Department of Health and Social Care [DHSC], 2021), many of whom live in care homes. The report states that pharmacists are optimally placed to undertake structured medication reviews in care homes. There is growing international interest in the place of pharmacists in care homes (Wright et al., 2020). In England, there is a policy initiative to recruit 6000 additional pharmacists into primary care by 2024 (National Health Service [NHS], 2019) and guidance that they would support structured medicine reviews in primary care and care homes (DHSC, 2021). Whilst increasing the number of pharmacists addresses resource challenges, there remains a need to address other barriers to tackling overprescribing. Barriers include complexities in decision‐making, concerns about potential adverse outcomes and the practitioner‐patient relationship (Anderson et al., 2019).

Deprescribing is defined as the process of identifying and stopping medicines with more risks than benefits (Reeve et al., 2015). It is key to reducing medication‐related harm and making it routine practice is central to the World Health Organisation's Third Global Patient Safety Challenge: Medicines without Harm (World Health organisation [WHO], 2017). Reactive deprescribing, which is stopping a medicine in response to an adverse outcome such as a side effect, is common practice across care settings (Scott, 2021). However, when medicines are deprescribed reactively, patient harm and the associated costs have already occurred (Scott et al., 2018). Proactive deprescribing is stopping a medicine before harm occurs. Whilst proactive deprescribing is an effective strategy to reduce medication‐related harm, it is also significantly more challenging than reactive deprescribing. This is because proactive deprescribing involves a difficult assessment of the chances of harm versus the chances of benefit (Scott et al., 2021).

Within the global drive to address polypharmacy in older people across all health settings, pharmacists have been identified as a key professional to support medication review alongside the physician (Mair et al., 2017). In some countries, pharmacists can undertake postgraduate training to become an independent prescriber. The acquisition of independent prescriber rights, coupled with their extensive understanding of how medicines work, their interactions and potential adverse effects, means pharmacists are ideally placed to deprescribe (Bleidt, 2019). The changing remit of pharmacists within community health settings is expanding their professional roles (International Pharmaceutical Federation [FIP], 2020). There is evidence that introducing pharmacists into care homes to address unnecessary or harmful prescribing can be effective (Alldred et al., 2016; Riordan et al., 2016). A systematic review found that pharmacist‐led services reduced the risk of falls but there was no clear evidence supporting outcomes in reducing hospitalisation and morality (Lee et al., 2019).

Our previous research identified the contextual factors and working relationships which enabled pharmacists to take a lead role in medicines management in care homes (Birt et al., 2021). The Care Home Independent Pharmacist Prescribing Study (CHIPPS) delivered an intervention that provided pharmacist‐independent prescribers, working with general practices, with dedicated time each week to undertake medicine management in named care homes (Bond et al., 2020a). Pharmacists received a bespoke two‐day training programme and mentor support to produce a portfolio of competencies (Wright et al., 2021). They reported this training increased their confidence in deprescribing with older people (Birt et al., 2022).

The CHIPPs study found substantial variation in the amount of deprescribing undertaken by pharmacists. For example, one pharmacist stopped 3 medicines over the six‐month trial, whilst another stopped 28. This variation was not associated with the duration of clinical experience but did appear to be associated with whether the pharmacist had previously worked in the associated general practice. Exploring the reasons for variation in deprescribing activity will enable the development of strategies which address barriers that lead to reduced activity and support enablers that lead to increased activity. This paper reports follow‐up interviews with pharmacists, GPs and care home managers who took part in the CHIPPS study between 2018 and 2020. The aim was to specifically explore primary care pharmacist, GPs and care home managers beliefs and practices of proactive deprescribing in care homes. Ethical approval was obtained from University of East Anglia, Faculty of Medicine and Health Sciences Ethical Review Committee.

## 2 METHODS

### 2.1 Recruitment and sampling

Participants were identified from those who had taken completed the CHIPPs intervention arm and included pharmacist‐Independent prescribers (PIPs, n = 23), General Practitioners (GP, n = 23) and care home managers (CHM, n = 38). All were provided with an electronic participant information sheet and invited by email to take part in a telephone or online interview. A purposive sampling framework was developed to sample for: location (urban and rural) and across Northern Ireland, Scotland and England (where the CHIPPS Study was conducted), representation across the three professional groups, and to ensure representation from pharmacists associated with high and low volumes of proactive deprescribing as found in the CHIPPS Study. Planned sample size was 30, with 10 participants from each professional group. Previous data collection with this group of professionals indicated they prepared for interview and were effective communicators able to draw on work examples and consider complexities of care, therefore it was highly likely this sample size would generate sufficient data to enable a credible analysis.

### 2.2 Data collection

A topic guide for semi‐structured interviews was developed based on the contextual factors identified within the earlier process evaluation as affecting deprescribing. Questions were also informed by the Theoretical Domains Framework (TDF) (Atkins et al., 2017) to elicit barriers or enablers to deprescribing behaviour as data would also inform future work on behaviour change strategies. The TDF synthesised 33 theories of behaviour and behaviour change and can be used to generate theory‐informed strategies in implementation research (McGowan et al., 2020). We used version 2 which has 14 domains and has undergone further validation from behavioural experts (Atkins et al., 2017). We also aimed to explore enablers to proactive deprescribing which may have already been implemented locally. The topic guide (see Appendix S1) was reviewed by pharmacy and general practice colleagues and by our patient and public advisory group, slight changes to language were made for clarity but no changes in the focus of questions. Interviews were offered virtually through Microsoft® Teams, Zoom or by telephone. All participants gave electronic informed consent prior to data collection.

### 2.3 Analysis

Data were analysed through reflexive thematic analysis (Braun & Clarke, 2019). Using an iterative process which started during data collection, it became evident that similar accounts were being offered, indicating we were achieving data saturation. After data familiarisation, data were coded inductively to examine deprescribing activity related to care home residents. We categorised the inductive coding and developed interpretative overarching themes to describe and define the factors which shape pharmacist‐led deprescribing in care homes. The transcripts were initially coded and categorised separately, and then explored for similarities and differences. Two overarching themes which encompassed the results from all professional groups were generated. To enhance the trustworthiness of the results, the researcher [LB] developed a coding framework from the first six interviews. The codes were discussed with the second researcher [SS], a pharmacist not previously involved in the intervention study. Codes were slightly refined, usually to increase detail. The coding framework and two interviews were conducted early in the sequence of interviews, and later the resulting themes were also discussed with the patient and public advisory group and the multidisciplinary research management team. No substantial changes were made. The COREQ tool (Tong et al., 2007) guided reporting (see Appendix S2).

## 3 RESULTS

Twenty‐nine interviews were conducted between May and August 2021, each lasting between 25 and 75 min. Table 1 provides a summary of participant demographic characteristics. Representation of professionals in this study was similar to that in the CHIPPs study process evaluation. There were professionals from the three included nations of the United Kingdom and variation in the pharmacist cohort between those who had high deprescribing activity (more than 20 deprescribing episodes in the 6‐month intervention) and those who had fewer (fewer than 10 deprescribing episodes in the 6‐month intervention).

**TABLE 1:** Participant demographic characteristics

Professional group	Location	Length of time qualified as an independent prescriber	Deprescribing interventions per resident	Type of General Practice (GP)	Type of care home
Pharmacist‐independent prescribers N = 16	Scotland n = 5 N Ireland n = 4 England n = 7	≤ 5 years n = 8 6–10 years n = 6 ≥ 11 years = 2	< 10 n = 5 10–19 n = 8 ≥ 20 n = 3	—	—
General Practitioners N = 6	Scotland n = 3 N Ireland n = 1 England n = 2	—	—	Rural n = 2 Urban n = 4	—
Care home managers N = 7	Scotland n = 3 N Ireland n = 2 England n = 2	—	—	—	Residential n = 2 With nursing n = 5

Pharmacists worked in a variety of contexts, with 12 working in a GP surgery they were employed by and four employed by others and working across multiple GP practices.

The two overarching themes generated from the interview data reflected the experiences of pharmacists and resonated with data from the GPs and care home staff. These were: (a) structures and systems affecting deprescribing, and (b) balancing risks when deprescribing.

### 3.1 Theme 1: Structures and systems affecting deprescribing

In this theme, it was evident that whether the pharmacist was employed in one GP surgery or working for several GP practices through the primary care network, there were working structures and systems. A work structure is the way individuals collaborate with each other to achieve work objectives, whilst a work system is a defined task which requires more than one person to complete it (Burke, 2017). Pharmacists worked within structures where leadership roles were often hierarchical and formal priorities and procedures appeared clearly defined and understood by those working within the structure. Relationships within the structure affected opportunities for deprescribing and created tensions between roles. Work systems within the GP practice‐enhanced opportunities for deprescribing and care home systems supported structured medicine review.

### 3.2 Deprescribing in a team work structure

The structures with primary care supported team activity related to deprescribing. There were accounts of multiple people being involved in deprescribing, either by directly undertaking the task or in supporting the fulfilment of the task. Pharmacist participants explained they saw deprescribing as a key part of their professional role:

It's an entirely appropriate role for us, we are the medicines experts. Pharmacist_2

GPs appreciate the role and skills of pharmacist reinforcing their place in the practice:

As a GP that we should do more medication reviews than we do, the pharmacists tend to be better at it. I actually really got a lot of good work out of working with [ PIP Name] and being able to deprescribe as part of a team. GP_13

Pharmacists faced competing demands on their time often running specialist clinics which meant the protected time to undertake deprescribing was not always there,

I think, more having protected time to do care home work maybe once a month, or once every two weeks, to have set days where you say. Pharmacist_23

Other healthcare practitioners were increasingly undertaking independent prescribing roles too, most usually Advanced Nurse Practitioners who visited care homes more regularly. Notably, a couple of pharmacists asserted that whilst healthcare professionals other than pharmacists have independent prescribing roles, they considered that non‐pharmacists may not have the confidence to deprescribe:

Do not think there's much confidence in reducing things from the nurse side of things. Pharmacist_1

Nonetheless, several pharmacists explained the nurses working in either the care home or the GP surgery would make them aware of possible deprescribing opportunities:

The manager was a nurse and she's very good you know if something isn't required she will contact us about stopping it. Pharmacist_3

However, there were accounts indicating that care home staff had different levels of clinical knowledge and confidence, and this might be an area where further training was needed to enhance deprescribing confidence in a care home. Building effective relationships with care home staff was identified as essential in creating a culture where deprescribing was acceptable:

You can't do that on just a one‐off visit, …it took a while to kind of build those relationships and for them to become more receptive to allowing you to you know make changes to the medicines. Pharmacist_4

Where the pharmacist worked with a pharmacy technician, they appeared to have a close working relationship. Pharmacy technicians often undertook audits of medications and medicine administration records in care homes, highlighting areas of concern to the pharmacist:

… working quite closely with the PCN (Primary Care Network) pharmacy technician so we're looking at deprescribing, … the technician they're going out to do some of the groundwork for the structured [medication review].. Pharmacist_6

Pharmacists who worked outside of a single GP practice, so working across multiple GP practices, appeared not to have developed relationships with an extensive team of healthcare professionals who were either identifying opportunities for deprescribing, or available for discussion of ideas. However, these pharmacists tended to work with a pharmacy technician and valued this support.

While generally there was a team ethos and approach, there were differing accounts. Two GPs stated that ultimate responsibility for prescribing and deprescribing activity was theirs rather than the pharmacists.

### 3.3 GP practice systems

Within the GP practice, pharmacists and GPs explained that signing off repeat prescriptions was a key time to review care home residents' medication. This provided a prompt to evaluate the number and nature of the medicines. Infection control practices during COVID‐19 had led to virtual reviews rather than face‐to‐face meetings. A limitation of virtual reviews was they did not provide the structures to build the relationships considered essential for safe and effective deprescribing:

One nursing home, they ring and leave messages for me, and I cannot overestimate/ underestimate the power of doing a face to face with them. … the benefits of taking time out to speak to the nurses about changes and what you were considering to do. Pharmacist_7

In a few GP practices, there was a dedicated administration team who dealt with all care home medicines issues. They were a conduit between care home and the pharmacist, facilitating communication.

### 3.4 Care home systems

Most care homes had 6‐ to 12‐monthly structured medication review meetings for each resident. These were usually multidisciplinary including GPs, pharmacists and nurses. Pharmacists and care home staff stated these formal reviews were an opportune time to consider deprescribing, especially as there were multiple different professionals available:

It does take time but that process [reviews] is really helpful because it's done as a team, and it's done with the pharmacist there, and there was a nurse from geriatric speciality team there, so it was really helpful and listening to their experiences, because I come at it with one focus, but they're coming at it with a medical background as well, which is really helpful. Care home manager_15

Participants noted that planning for these reviews took time, but that this was easier now that many meetings were virtual rather than in person, slightly contradicting statements about the power of face‐to‐face contact.

Another regular system in care homes was the admission procedure for a new resident. While nearly all participants mentioned that older people in the community often have polypharmacy there were differing accounts of whether deprescribing should be a task in the new admission procedure. Several pharmacists stated the resident needed time to settle before any medicine changes were made, but others explained they would quickly stop medicines they considered non‐essential: usually creams and laxatives.

### 3.5 Theme 2: Balancing risks when deprescribing

All participants stated there was potential for unintended consequences from proactive deprescribing. Pharmacists and GPs described making complex decisions around the risk of deprescribing. These were centred on: knowing the person and the frailty of the resident and whether deprescribing aligned with published medical guidance.

Proactive deprescribing remained a team effort as the risk was mitigated when care home staff were able to monitor residents for adverse effects.

### 3.6 Knowing the patient

All participants spoke of the need to ‘know’ a resident before trying to deprescribe. This was associated with the potential for detrimental impact on behaviour or worsening clinical symptoms:

We don't want just to take someone in and stop medications when we don't know why they're on them or the reasons behind it. Care home manager_16

Several pharmacists stated that deprescribing required a multi‐layered approach; there was a need not only to look at the medicines the resident was taking but also the clinical reasons and history of the medicine. This could be done in stages and several suggested that on admission to a care home, an initial review was needed, only stopping those medicines that were no longer being taken (as reported by the resident or family) or low‐risk items such as topical products, for example lotions. There were several reasons offered for not making sudden deprescribing decisions on admission:

Residents arrive with the little packet of tablets in those dosette boxes, and that's all you know. You don't know what's wrong with them. You don't know why they're on it. You don't know when it started. You basically just check with the nursing home what are they on you're fingers crossed, quite bluntly, and hoping that you're OK with giving them … we don't do anything drastic to start with because we don't want to destabilise them, and they'll already have been through trauma, being transferred [into care home]. Pharmacist_20

More complex proactive deprescribing happened when care home staff knew a resident's behaviour and needs, and clinical records had been obtained enabling a more detailed systematic medication review to be undertaken. It was usually at this point that deprescribing of cardiovascular and antipsychotic medications was considered. This comprehensive review was a time‐consuming activity, but all pharmacists stated they needed to be able to provide and record their clinical reasoning for deprescribing as this potentially mitigated risk to their professional status:

I am methodical go through each of the drugs to find out why they were started, if it is very historic, I will go back as far as reasonably possible to identify why it was started ‐ might be going through clinic letters, hospital discharges trying to piece the jigsaw puzzle together. Then it's a case of checking clinically if it's still appropriate, so are their observations within range, are their blood tests up to date. Then having a chat to the patient “how are you getting on with your medication? because often we overlook the patient. Pharmacist_29

When this type of structured medication review was possible, many pharmacists reported examples where medicines prescribed many years ago no longer had a clinical indication and were stopped to the benefit of the resident. There were also positive impacts on resources, namely medication cost and staff time in ordering and dispensing.

### 3.7 Frailty of resident

Many participants spoke about having confidence to make decisions on deprescribing when the resident was frail. Usually, this was in relation to a lack of clinical evidence on a benefit such as with the prescribing of statins in people approaching the end of life. When the resident was frail, the balance of risk favoured stopping such medications:

I suppose it comes down to confidence, primarily people are scared to deprescribe because they are scared of consequences and us GPs are very risk‐averse…but I think it is easier when you are dealing with patients in their 80 s and 90 s they are frail and you think what could that medication actually be doing for them let us have a go and stop it and see how they get on. GP_12

Pharmacists and GPs explained that the risk of deprescribing was mitigated when care home staff were able to monitor the resident when medicines were stopped or titrated down. This facilitated the timely identification of potential side effects:

…feel more confident in the care home environment because they've got nurses to sort of monitor the results of the deprescribing and see if things are changing. GP_11

However, there was always a concern about potential harm arising from deprescribing and participants spoke of trying to seek resident and family agreement. One pharmacist spoke of deciding whether a proactive deprescribing action was worth pursuing:

Some things are not worth fighting about because if something happens to that patient you'd be the first one in court. It would be different if I thought actually it's [the medicine] more harmful. Pharmacist_29

While all participants acknowledged risks in deprescribing, they also recounted that medicines could be restarted and changes in behaviour following a dose reduction, and stopping, may not necessarily be causal in nature. This account by a care home manager illustrates the risk and complexity of deprescribing:

It's sometimes trying to know whether the behaviour is because of their dementia. Whether the behaviour is because of pain. Whether it's because of infection. There are challenges trying to rule out different things and it can be time consuming, although the benefit [of deprescribing] is amazing it can be a lot of work. Care home manager_15

### 3.8 Professional competence

Pharmacists and GPs described made complex decisions around the risk of deprescribing and these were centred on professional competence and alignment with guidance:

As a pharmacist, you're very much taught to live in the black and the white in terms of your decisions. But I think certainly working in primary care, I've been aware of the need to make decisions in the grey. You need to think about your clinical reasoning in weighing things up. But I still like that comfort of having something to guide or to help facilitate your decision‐making, and I just think it provides consistency of approach as well. Pharmacist_10

Several participants explained certain medicines had a low risk of severe adverse effects if stopped and that they were often over‐prescribed. For medicines such as vitamins, topical products and laxatives, pharmacists and GPs were happy to deprescribe without extensive consideration of clinical history or involvement from the resident's family.

Depending on their prescribing speciality, pharmacists differed in what they felt competent to deprescribe without the involvement of the GP. When a medicine had recently been prescribed within secondary care there was a general reluctance to stop it without consulting the prescriber. However, even when the pharmacist was not confident to deprescribe for a person who recently had heart surgery, they still compiled all clinical information to support a deprescribing decision, suggesting this saved GP time:

I did all the investigating to work out why was this [medicine] started in that context and what has changed and I went and presented the stuff I had found out to the GP. It would have taken the GP a long time to work all that out, but I was able to present it to them and they trust me enough you know having worked with them for a while and the trust point are so important. Pharmacist_1

### 3.9 Drawing on deprescribing guidance

Pharmacists and GPs stated that while national alerts about medicines from government sources, or through more informal deprescribing groups, were a trigger for reviewing polypharmacy and identifying care home residents on such medicines, there was still uncertainty and this made proactive deprescribing more complex:

it feels like it [proactice deprescribing] takes more energy and is slightly more complicated, and I think that's because of guidelines. So, starting a medication, I've got a guideline that will tell me which medications to consider. If I'm stopping medications there's less structure. GP_14

Alerts and guidance on deprescribing were not always relevant or tailored to older people and this led pharmacists to explain guidance needed to be balanced against individual patient risk:

It's accountability and responsibility for those decisions, and I don't think there's an appreciation that the suggestions that are being made have consequences and it's very easy to read guidance and best practice, and there's so much information out there but it still always comes down to that individual patient and their experiences, and their overall health. Pharmacist_5

Amongst pharmacists there was a desire for more support for deprescribing, both in guidelines tailored for older people and in informal support from peers:

Building a network would be really helpful, education and training it would be nice … Hopefully it will increase because at one point I felt like I was on my own doing this. Pharmacist_29

## 4 DISCUSSION

The central role of pharmacist‐independent prescribers in managing medicines and deprescribing within the primary care team has been reinforced by results from this interview study. Our findings illustrate the complexity of clinical decision‐making that lies behind deprescribing a medicine. While there were concerns about the risks of proactive deprescribing for care home residents, there was overwhelming agreement that addressing problematic polypharmacy outweighed any perceived risks.

Team structure is important for effective working practices (Ji & Yan, 2020) and we found that when pharmacists were employed in GP surgeries communication was effective and professional understanding of clinical expertise and distinct roles highly evident. Teamwork supported proactive deprescribing. This resonates with evidence that pharmacists working in care homes, who were also well‐embedded into the wider primary care multidisciplinary team made more interventions than those who are not working in an established team (Cherubini et al., 2016). It is likely that shared digital systems and shared working spaces support clinical discussions (Kings Fund, 2020). However, national policies may be focused on the more general deployment of the pharmacist within wider community work structures and Furter evidence is required to understand whether the pharmacists may be more effective if employed directly with one medical practice rather than a within a group.

Our study identified that a lack of clearly defined responsibilities and protected time to undertake deprescribing could prevent the activity from happening. In the United Kingdom, the NHS Long‐Term plan commits to increasing the number of pharmacists in primary care and advocates for the pharmacist to receive clinical training to deliver structured medication reviews, improve medicine safety, support care homes and run practice clinics. (NHS, 2019). The National Overprescribing Review recommends pharmacists have a major role in medicines management (DHSC, 2021) and the Framework for Enhanced Health in Care Homes recommends annual multi‐disciplinary medication reviews (NHS, 2020). Yet, there are suggestions that mandatory strategies may be more effective than guidance or recommendations at supporting medicine review practices (Spinewine et al., 2021). The impact of national guidance will need to be monitored to see if legislation is needed to support evidence‐based guidance.

There is growing international evidence that involving a pharmacist in care home resident medication review whether as the prescriber or in collaboration with a GP increases deprescribing of medicines and this has associated cost savings (Baqir et al., 2017; Kua et al., 2019; Riordan et al., 2016; Sadowski et al., 2020). However, our results indicate that the focus on quantitative measurable outcomes in pharmacist intervention studies, such as reduction in falls and mortality, may miss the more resident‐focused, and system‐orientated outcomes which may arise from pharmacists working in care homes. A consistent narrative in our data was that deprescribing had positive person‐centred outcomes for the care home resident, care home staff and more widely for organisational resource use. While there was talk of regular medicine reviews in our study participants did not appear to have clearly defined structures in place for reviews, in part this may have been due to the work environment in the post‐COVID‐19 environment. Nonetheless, there remains a need for further research to understand the optimum times for medication reviews as Chao and MacDougall found limited evidence of cost‐effectiveness and clinical efficacy in 3 monthly reviews (Chao & MacDougall, 2019).

Although our results indicate that experientially and cognitively participants were aware of the benefits of deprescribing medicines that were no longer needed or potentially harmful deprescribing was still considered to be potentially risky. Participants spoke of an absence of clear guidance with only five saying they used the STOPP tool (Screening Tool of Older Persons' potentially inappropriate Prescriptions) criteria (O'Mahony et al., 2015). In part, this may be addressed by greater access to the American Geriatrics Association (2019); Beers criteria® for Potential Inappropriate Medicine use in Older People (2019) which encompass people over 65 years in institutional setting. With the challenges of an ageing population and national shortage of general practitioners, there is a need for greater focus on deprescribing within pharmacist training (Clark et al., 2020).

### 4.1 Strengths and limitations

The diverse sample provided representation from across the UK and from different primary care stakeholders increasing the likelihood that data have relevance to a variety of primary care settings. However, representation from Northern Ireland was limited, although this was a smaller pool of potential participants as the process evaluation only had five intervention sites in Northern Ireland (Bond et al., 2020b). A limitation of the research is the pharmacists had previously been exposed to a pharmacist‐led medicines management intervention (Wright et al., 2021), therefore they may have been more aware, competent and enthusiastic about proactive deprescribing than practitioners who had not had such intensive exposure. Pharmacist participants did reference the learning they had experienced in the previous intervention study and how they now applied that to practice. However, several pharmacists had moved to new roles and some stated that in their current role, they had less time for structured medication reviews. It was over 18 months since the intervention study so there may have been some recall bias, but questions were focused on current practice. A limitation of the study design was the absence of resident and family voices. Future work should include resident's voices, so as to empower them to have conversations about deprescribing (Ailabouni et al., 2022). Across healthcare sectors, the public are willing to consider stopping a medicine if it is recommended by a doctor (Weir et al., 2022). There may need to be educated to ensure the public is aware that deprescribing is a professional role of pharmacists.

### 4.2 Implications for practice

Polypharmacy and its adverse outcomes in older care home residents is extremely well documented. Internationally primary care practice is seeking to use the skills of pharmacists in long‐term care. Findings from our study indicate that pharmacists see themselves in this role and this is generally endorsed by the other stakeholders, in particular, GPs and care home staff. Other HCPs within the primary care team such as nurses and pharmacy technicians have an essential role in supporting the pharmacist by providing detailed clinical information about the resident, discussing complex clinical cases and monitoring residents' condition after deprescribing. Deprescribing by either a GP or pharmacist has been identified as a key activity for reducing medication errors (National Care Forum, 2019) and medication‐related adverse events. The shortage of GPs indicates that now is the right time to endorse and support the primary care pharmacist role in care home medicines management. Yet, there remains a need for empirical evidence on the contextual factors, barriers and enablers which shape deprescribing in care homes (Sawan et al., 2020).

## 5 CONCLUSIONS

In summary, pharmacists, GPs and care home managers were in favour of deprescribing in order to reduce problematic polypharmacy for care home residents. Primary care pharmacists have the clinical skills and professional competence to undertake structured medication reviews in older people in care homes. Primary care team structures can support deprescribing by pharmacists, but it was acknowledged that deprescribing was a complex clinical activity with some inherent risks to resident's wellbeing. Nonetheless, all professionals strongly stated that deprescribing was an essential feature to improve care of older people in care homes in most cases positive outcomes were reported.

## AUTHOR CONTRIBUTIONS

DW, CB, RH, DA, CH and SS conceived the overall research design and provided commentary on the progress of the data collection and analysis. LB undertook qualitative data collection supported by JB. LB and SS undertook analysis supported by JB. All authors provided commentary on the data analysis helping to refine the final themes. LB, DW, JB and SS produced the manuscript. All authors commented on versions of the manuscript, and all agree to the final version.

## CONFLICT OF INTEREST

The authors did not have any competing interests.

## FUNDING STATEMENT

This research is funded by the National Institute for Health Research (NIHR) Policy Research Programme (project reference NIHR202053). The views expressed are those of the authors and not necessarily those of the NIHR or the Department of Health and Social Care.

## Supporting information


Appendix S1

Appendix S2

## Data Availability

Data available on request from the authors.
