# Supplementary material for: Enhancing deprescribing: A qualitative understanding of the complexities of pharmacist‐led deprescribing in care homes
Source: Health Soc Care Community. 2022 Nov 6;30(6):e6521–31. doi: 10.1111/hsc.14099 (PMC10100492; doi:10.1111/hsc.14099)
Supplement: Supplementary file 1 — Appendix S1 [file HSC-30-e6521-s001.docx]

Interview Topic Guide

**Introduction**

**Before we start I would like to clarify that by deprescribing we are talking about activity that leads to a medication being stopped, or reduced with the intention of alleviating potential harm. We are particularly interested in proactive deprescribing rather than reactive deprescribing where there has been clinical need**

| **Anchor question** | **Potential probes** | **Links to TDF** |
| --- | --- | --- |
| **How does deprescribing fit in with your role?**  **What do you think about deprescribing being a part of your role?** | GP: opportunities for proactive deprescribing  CH staff: opportunities to consider proactive deprescribing  PIPs: opportunities for proactive deprescribing, competence | Professional identify  Beliefs about capabilities |
| **Who else do you work with when you deprescribe?** | Whose opinions seek: family, resident, care home staff, GP , other practice staff, secondary care experts, community pharmacist, others | Social/professional roles  Social influences |
| **Please tell me about your thoughts on proactive deprescribing in care homes?** | What are benefits of proactive deprescribing (resident, DBI, for pharmacist, GP, CH staff more global benefits  What might be disadvantages (risks, harms, missed opportunities for alternative activities)  Draw out any practice examples | Knowledge  Motivations and Goals  Optimism  Beliefs about consequences |
| **What are the things that help you to deprescribing in care homes?** | Any social influences (seen it work, having, trust in deprescribing professional)  Are there any environmental factors or resources which positively affect deprescribing (Prescence of pharmacist or GP, time, how often medicines routinely reviewed)  Are there any attitudinal factors which positively affect deprescribing (staff, family, resident)  Are there any governance or policy factors which positively affect deprescribing | Beliefs about capabilities and consequences  Environmental context and resources    Social influences, professional roles, Optimism |
| **What are the things that could help you do MORE deprescribing in care homes (**Pick up follow ups depending on above responses) | Any social influences (seen it work, having, trust in deprescribing professional)  Are there any environmental factors or resources which positively affect deprescribing (Prescence of pharmacist or GP, time, how often medicines routinely reviewed)  Are there any attitudinal factors which positively affect deprescribing (staff, family, resident)  Are there any governance or policy factors which positively affect deprescribing | Beliefs about capabilities and consequences  Environmental context and resources    Social influences, professional roles, Optimism |
| **Please tell me what are the challenges that stop you from deprescribing in care homes?  (tailor depending on responses above)** | Any social influences (beliefs about only happen end of life, may increase behaviour which challenge)  Are there any environmental factors or resources which affect deprescribing (Prescence of pharmacist or GP, time, how often medicines routinely reviewed)  Are there any organisational governance or policy factors which affect deprescribing  Are there any wider governance or policy factors which affect deprescribing  Any different challenges for the team (PIP, GP CH depending on interviewee  Within the team, organisation and beyond | Social Influences  Beliefs about consequences  Environmental context and resources  Professional roles |
