# Supplementary material for: Enhancing deprescribing: A qualitative understanding of the complexities of pharmacist‐led deprescribing in care homes
Source: Health Soc Care Community. 2022 Nov 6;30(6):e6521–31. doi: 10.1111/hsc.14099 (PMC10100492; doi:10.1111/hsc.14099)
Supplement: Supplementary file 2 — Appendix S2 [file HSC-30-e6521-s002.docx]

**Supplementary file 2 Consolidated criteria for reporting qualitative studies (COREQ): 32-item checklist**

Developed from:

Tong A, Sainsbury P, Craig J. Consolidated criteria for reporting qualitative research (COREQ): a 32-item checklist for interviews and focus groups. *International Journal for Quality in Health Care*. 2007. Volume 19, Number 6: pp. 349 – 357

| **No. Item** | **Guide questions/description** | **Reported on Page #** |
| --- | --- | --- |
| **Domain 1: Research team and reﬂexivity** |  |  |
| *Personal Characteristics* |  |  |
| 1. Inter viewer/facilitator | Which author/s conducted the interview or focus group? | LB |
| 2. Credentials | What were the researcher’s credentials? E.g. PhD, MD | PhD extensive experience in qualitative methods |
| 3. Occupation | What was their occupation at the time of the study? | Researcher |
| 4. Gender | Was the researcher male or female? | Female |
| 5. Experience and training | What experience or training did the researcher have? | PhD and 10 years post-doctoral experience |
| *Relationship with participants* |  |  |
| 6. Relationship established | Was a relationship established prior to study commencement? | Some participants had been interviewed in previous study |
| 7. Participant knowledge of the interviewer | What did the participants know about the researcher? e.g. personal goals, reasons for doing the research | Participants were aware the study was a follow on from the intervention study and the particular focus was on proactive deprescribing. This was in the participant information sheet |
| 8. Interviewer characteristics | What characteristics were reported about the inter viewer/facilitator? e.g. Bias, assumptions, reasons and interests in the research topic | Reflexive iterative analysis was undertaken the research does not work in primary care and has no vested interest in the outcomes page 5 |
| **Domain 2: study design** |  |  |
| *Theoretical framework* |  |  |
| 9. Methodological orientation and Theory | What methodological orientation was stated to underpin the study? e.g. grounded theory, discourse analysis, ethnography, phenomenology, content analysis | The reported study uses thematic analysis Page 5 |
| *Participant selection* |  |  |
| 10. Sampling | How were participants selected? e.g. purposive, convenience, consecutive, snowball | Purposive sampling of a defined bou8nded cohort all invited to interview and sampling framework in place Page 4 |
| 11. Method of approach | How were participants approached? e.g. face-to-face, telephone, mail, email | Contacted by email Page 4 |
| 12. Sample size | How many participants were in the study? | 29 |
| 13. Non-participation | How many people refused to participate or dropped out? Reasons? | 39 no responses to email invitation 3 replied not interested, 7 left positions 2 emails undeliverable |
| *Setting* |  |  |
| 14. Setting of data collection | Where was the data collected? e.g. home, clinic, workplace | Data collected virtually Page 5 |
| 15. Presence of non-participants | Was anyone else present besides the participants and researchers? | No |
| 16. Description of sample | What are the important characteristics of the sample? e.g. demographic data, date | Table 1 reports profession location and length of time been a prescribing pharmacist |
| *Data collection* |  |  |
| 17. Interview guide | Were questions, prompts, guides provided by the authors? Was it pilot tested? | Not pilot tested but reviewed by experts and PPI Page 5 |
| 18. Repeat interviews | Were repeat inter views carried out? If yes, how many? | NO |
| 19. Audio/visual recording | Did the research use audio or visual recording to collect the data? | Audio recording Page 5 |
| 20. Field notes | Were ﬁeld notes made during and/or after the interview or focus group? | Researcher reflexive memos kept Page 5 |
| 21. Duration | What was the duration of the inter views or focus group? | 25 to 75 minutes Page 5 |
| 22. Data saturation | Was data saturation discussed? | Yes Page 15 |
| 23. Transcripts returned | Were transcripts returned to participants for comment and/or correction? | No |
| **Domain 3: analysis and ﬁndings** |  |  |
| *Data analysis* |  |  |
| 24. Number of data coders | How many data coders coded the data? | Primarily LB but codes discussed and developed with wider team Page 5 |
| 25. Description of the coding tree | Did authors provide a description of the coding tree? | No |
| 26. Derivation of themes | Were themes identiﬁed in advance or derived from the data? | Inductive analysis Page 5 |
| 27. Software | What software, if applicable, was used to manage the data? | NVivo Page 5 |
| 28. Participant checking | Did participants provide feedback on the ﬁndings? | The results were shared with a diverse research team for feedback Page 5 |
| *Reporting* |  |  |
| 29. Quotations presented | Were participant quotations presented to illustrate the themes/ﬁndings? Was each quotation identiﬁed? e.g. participant number | Yes in results 6 -13 |
| 30. Data and ﬁndings consistent | Was there consistency between the data presented and the ﬁndings? | Yes |
| 31. Clarity of major themes | Were major themes clearly presented in the ﬁndings? | Yes |
| 32. Clarity of minor themes | Is there a description of diverse cases or discussion of minor themes? | Yes discussion of minority of responses which indicated concern in proactive deprescribing |
